# Physical Exercise and Brain Functions in Older Adults

**DOI:** 10.1155/2013/197326

**Published:** 2013-09-17

**Authors:** Louis Bherer, Kirk I. Erickson, Teresa Liu-Ambrose

**Affiliations:** ^1^PERFORM Centre, Concordia University, Montreal, QC, Canada; ^2^Research Center, Institut Universitaire de Gériatrie de Montréal, Montreal, QC, Canada; ^3^Department of Psychology, University of Pittsburgh, Pittsburgh, PA, USA; ^4^Department of Physical Therapy, University of British Columbia, Vancouver, BC, Canada; ^5^Brain Research Centre, University of British Columbia, Vancouver Coastal Health Research Institute, Vancouver, BC, Canada

Studies suggest that regular physical activity can help maintain and enhance brain functions in older adults. However, we still do not understand how physical activity impacts the rate of cognitive decline. One major issue is whether physical activity broadly defined (i.e., activity that is part of one's daily life involving bodily movements and the use of skeletal muscles) or structured exercise (i.e., physical activity that is planned, structured, and purposive to improve physical fitness) leads to the same benefits in preventing age-related cognitive decline. More studies are needed to appreciate the level of change or protection provided by physical activity, the basic mechanisms by which this change occurs, and whether physical activity can be beneficial despite chronic medical conditions and neurological or geriatric syndromes. 

This special issue presents original research results that bring additional support to the notion that physical exercise is an efficient nonpharmaceutical approach that can be used to enhance and maintain cognitive functions in healthy older adults and patients suffering from mild cognitive impairment. In a paper, we propose a brief review of the main impacts of exercise on cognition in older adults, frail patients, and those with mild cognitive impairment and dementia. Another paper of this special issue brings important knowledge in this regard. L. S. Nagamatsu et al. showed that physical activity helps improve verbal and spatial memory in older adults with probable mild cognitive impairment. In this study, eighty-six women aged 70–80 years with subjective memory complaints completed one of three interventions for 6 months: resistance training, aerobic training, or balance and tone (control). Both exercise groups showed significant improvements in memory performance, which was not observed in controls.

An original study of this special issue explored the benefits of swimming on cognition in older adults. A. Abou-Dest et al. compared three groups of sixteen volunteer participants (young adults, sedentary older adults, and older adults who regularly practice swimming) on a battery of cognitive tasks. They reported that in older adults, regular swimming was related to better performance on executive functions but not on information processing speed. The selective benefit of exercise on executive control tasks was also reported after only 3 months of exercise intervention in a study of this special issue by D. Predovan et al. compared to controls, the training group showed significant improvements in physical capacity and enhanced Stroop performance, but only in the inhibition/switching condition, and the increase in aerobic capacity induced by the training regimen correlated negatively with reaction time in the inhibition/switching condition of the Stroop task at posttest. Importantly, the reported gains in cognitive performance were observed after only three months of physical training. The complex interaction between bodily exercise and cognition also calls into question the impact that mobility and gait might have on cognition in older adults. P. Plummer-D'Amato et al. addressed this issue in a contribution to this special issue. Studying how gait difficulty and cognitive task difficulty impact cognitive-motor interference in aging, they showed that gait task difficulty influences dual-task effects on gait speed, especially in older adults, and that this effect is influenced by the difficulty of the cognitive task.

Another paper of the special issue brings up important issues on the potential moderators of physical activity on brain functions. R. L. Leckie et al. demonstrated how genes (APOE, BDNF, and COMT) along with dietary omega-3 fatty acid and docosahexaenoic acid (DHA) are potential moderators of the effect of physical activity on brain health. R. L. Leckie et al.'s proposal calls for further studies on the role of genes and dietary factors in the relationship between physical exercise and cognitive functions in older adults populations. All together, the studies published in this special issue bring additional scientific support to the notion that physical activity and exercise are a promising approach to alleviate the age-related impact on the body and mind. By doing so, they also support the promotion of health policies that should target inactivity in individuals of all ages and with any medical condition who are able to safely participate in physical activity. 

